# A predictive model for hospitalization and survival to COVID-19 in a retrospective population-based study

**DOI:** 10.1038/s41598-022-22547-9

**Published:** 2022-10-28

**Authors:** Alejandro Cisterna-García, Antonio Guillén-Teruel, Marcos Caracena, Enrique Pérez, Fernando Jiménez, Francisco J. Francisco-Verdú, Gabriel Reina, Enrique González-Billalabeitia, José Palma, Álvaro Sánchez-Ferrer, Juan A. Botía

**Affiliations:** 1 Departamento de Ingeniería de la Información y Las Comunicaciones, Universidad de Murcia, Murcia, Spain; 2 Departamento de Informática, Servicio Murciano de Salud, Comunidad Autónoma de la Región de Murcia, Murcia, Spain; 3 Servicio de Microbiología. Clínica, Universidad de Navarra, Pamplona, Spain; 4 Department of Medical Oncology, Hospital Universitario, 12 de Octubre, Madrid, Spain; 5 Department of Biochemistry and Molecular Biology-A, Faculty of Biology, Regional Campus of International Excellence “Campus Mare Nostrum”, University of Murcia, Campus Espinardo, 30100 Murcia, Spain; 6 Murcia Biomedical Research Institute (IMIB-Arrixaca), 30120 Murcia, Spain; 7 Universidad Católica San Antonio de Murcia-UCAM, Murcia, Spain; 8 Department of Neurodegenerative Disease, Institute of Neurology, University College London, London, UK

**Keywords:** Prognosis, Computational science, Statistics

## Abstract

The development of tools that provide early triage of COVID-19 patients with minimal use of diagnostic tests, based on easily accessible data, can be of vital importance in reducing COVID-19 mortality rates during high-incidence scenarios. This work proposes a machine learning model to predict mortality and risk of hospitalization using both 2 simple demographic features and 19 comorbidities obtained from 86,867 electronic medical records of COVID-19 patients, and a new method (LR-IPIP) designed to deal with data imbalance problems. The model was able to predict with high accuracy (90–93%, ROC-AUC = 0.94) the patient's final status (deceased or discharged), while its accuracy was medium (71–73%, ROC-AUC = 0.75) with respect to the risk of hospitalization. The most relevant characteristics for these models were age, sex, number of comorbidities, osteoarthritis, obesity, depression, and renal failure. Finally, to facilitate its use by clinicians, a user-friendly website has been developed (https://alejandrocisterna.shinyapps.io/PROVIA).

## Introduction

The virus responsible for Coronavirus disease 2019 (COVID-19), the severe acute respiratory syndrome coronavirus 2 (SARS-CoV-2), is a highly transmissible and pathogenic betacoronavirus that appeared in late 2019 in Wuhan, China^1^. As of February 2022, it has had a tragic effect on the world’s population health resulting in more than 5,8 million deaths and 412 million cases worldwide, becoming the most important global health crisis since the era of the influenza pandemic of 1918^2,3^. The symptoms of COVID-19 are wide and may include fever, cough, fatigue, gastrointestinal issues, sore throat, anosmia, hyposmia, and neurological symptoms^4–7^. Some of these symptoms can persist after recovery, particularly fatigue and dyspnea^8^. The mortality rate of COVID-19 worldwide is around 1.5% since the pandemic began until February 2022 according to the World Health Organization (WHO). Although, we are aware of the existence of people more likely to develop a critical illness and eventually die^9^. In addition, it has been proven that vaccines reduce COVID-19 death rates^10,11^. For instance, the latest Centers from Disease Control and Prevention (CDC) release from the United States concluded that unvaccinated people have more COVID-19-associated death risk (mortality rate is around 1.39%) than fully vaccinated people (mortality rate is around 0.78%) with or without booster doses^12^. Due to the high contagiousness and rapid spread of SARS-CoV-2, many countries have to manage intense periods of the disease, which are known as waves^13^. In these periods, hospital resources, intensive care unit (ICU) capacity and healthcare system saturation can contribute to the increase in case fatality^14^. Hence, clinical management of patients, quick risk stratification strategies, and optimization of the use of resources are important to reduce the fatality rate^15,16^.

Electronic medical records (EHR) are one of the main resources to improve the way we approach patient management and move toward a more efficient triage of COVID-19 patients. Thus, patient demographic and health data available through healthcare systems have been used for COVID-19 patient prognosis and evolution through the use of semi-automated Artificial Intelligence (AI) systems. For example, a machine learning-based XGBoost model has been developed to predict patient mortality rates more than 10 days in advance with an accuracy of about 90%, using three biomarkers as main indicators for predicting COVID-19 prognosis, lactate dehydrogenase (LDH), high-sensitivity C-reactive protein (hs-CRP), and lymphocyte count^17^. A higher ROC-AUC (0.96) was obtained with clinical data of patients at admission using four machine learning methods including logistic regression, support vector machine, decision tree with gradient boosting, and neural network^18^. Using LASSO and a predictive equation with binary logistic regression based on pre-existing comorbidities and demographic data it was concluded that these variables demonstrated a good ability to discriminate severe from non-serious outcomes using only this historical information with an AUC of 0.76^19^. A further study developed models based on machine learning with different techniques, LASSO, novel univariate and pairwise, but concluded that no model was able to outperform a model based solely on age, where age had an AUC of 0.85 and balanced accuracy of 0.77^20^. Another model was able to predict the risk of hospital/ICU admission and death already at diagnosis with a ROC-AUC of 0.902 by focusing only on a limited number of comorbidities and demographic variables, such as age, sex, and BMI^21^. In all of the above cases, the healthcare dataset is imbalanced, as individuals with the most severe disease episodes are in the minority, so a supervised machine learning approach aimed at modeling them will suffer from imbalance. Moreover, the predictors considered for such studies are difficult to obtain. For example, LDH, albumin (ALB), blood urea nitrogen (BUN), hs-CRP, and lymphocytes require the use of blood or urea tests; or else the taking of measurements with specific physical devices, as is the case for BMI, temperature, and oxygen saturation. Therefore, these models are far from being realistically usable for early triage of patients in times of emergency oversaturation.

Our study presents a technique specially designed to address imbalanced problems applied to streamlining and improving the triage of COVID-19 patients according to their age, sex, and comorbidities, based on data readily available within the Regional Health System of the Region of Murcia, located in southeastern Spain. This allows us to perform a local study in which data are organized in five different sources, including information on clinical history, hospitalization services, symptoms, vital signs, performed treatments in more than 100,000 COVID-19 patients with diagnosis dates ranging from January 4th, 2020 to February 4th, 2021. It must be pointed out that this study does not focus on the effects of vaccination. Note that just 1.4% of the total population of Spain was vaccinated by the date that the last patient was enrolled in our study (https://ourworldindata.org/covid-vaccinations?country=ESP). This leaves us with just approximately 402 subjects with a vaccine. The technique for dealing with imbalance consists of dividing the original problem into *p* subproblems, where each one will have a perfectly balanced data set associated with it, formed by samples from the original set. Using this reasoning, it is possible to build an ensemble logistic regression model that allows obtaining ROC-AUC of 0.94 to predict the final condition of the patient (discharge or deceased) similar to complex models that combine several machine learning methods, and similar or higher than those that combine much more complex data based on laboratory or care techniques.

## Results

### Description and differences of the different types of COVID-19 patients in our dataset

**Figure 1:**
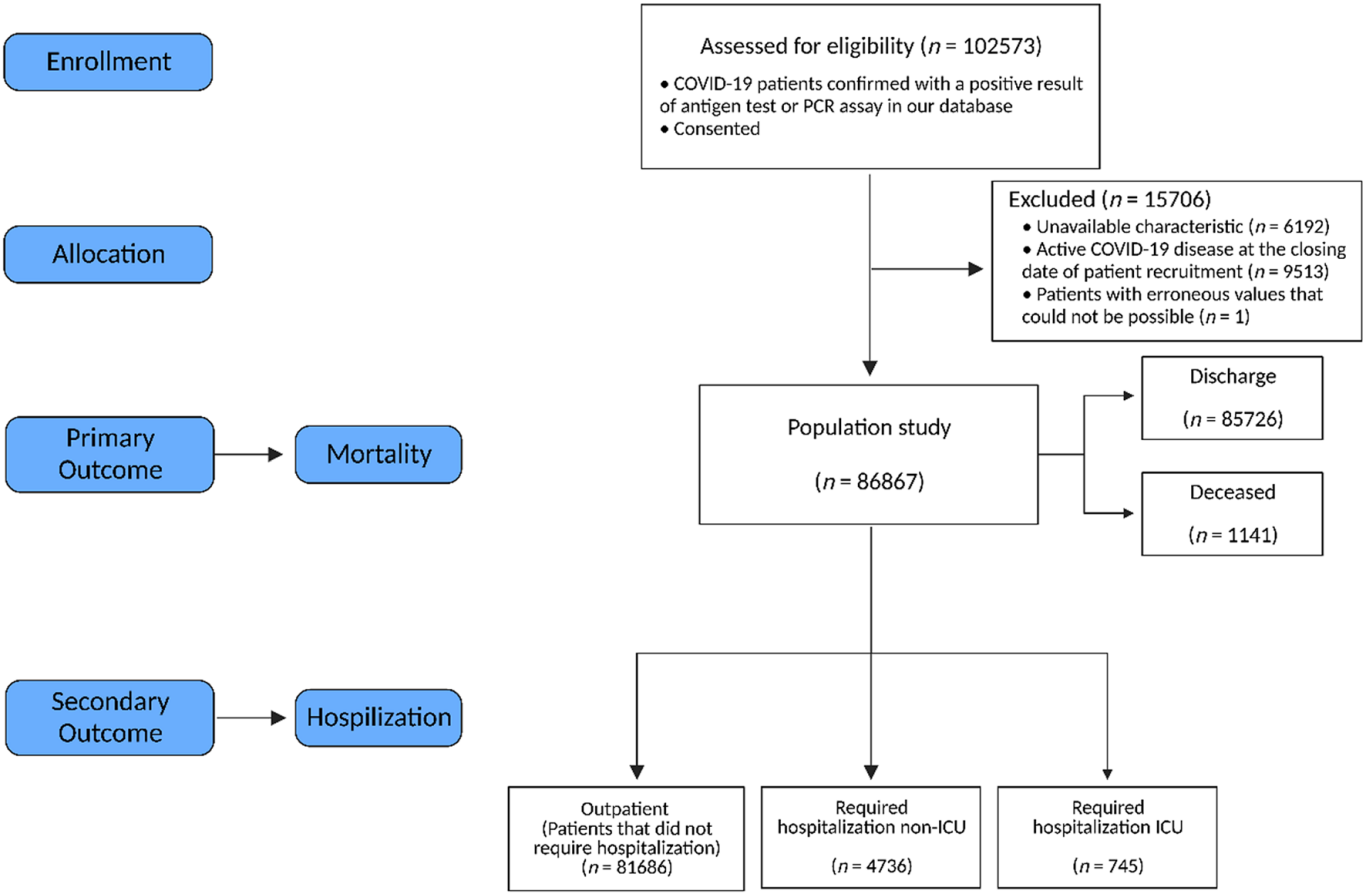
CONSORT diagram. Flow diagram of the enrollment of subjects, disposition status, and how they are analyzed in the study.

The exploratory analysis of the data from 86,867 COVID-19 patients in a region located in the southeast of Spain (Region of Murcia) allowed stratifying the database obtained by age, sex, and specific comorbidities (Table 1), following the flow chart for the cohort shown in Fig. 1. Among the cases studied, 93.7% were outpatients (N = 81,386), 5.4% were hospitalized non-ICU (N = 4736) and less than 0.85% were patients admitted to the ICU (N = 745). The most common symptoms among patients were cough (49.9%), followed by headache (38.3%) and myalgia (36%) (Supplementary Table S1). Using the data in Table 1 we can identify the different prototypes of patients with COVID-19.

**Table 1:** Demographic characteristics, comorbidities, and final outcome of different types of COVID-19 patients.

Characteristics	Outpatient	Hospitalized (non-ICU)	ICU
Number of individuals (N)	81,386	4736	745
Age median (IQR)	38.00 (22.00, 52.00)	62.00 (47.00, 79.00)	62 (52.00, 71.00)
**Gender**			
Male (%)	38,200 (46.94%)	2450 (51.73%)	524 (70.34%)
Female (%)	43,186 (53.06%)	2286 (48.27%)	221 (29.66%)
**Comorbidities**			
Number of Chronic diseases median (IQR)	2.00 (1.00, 4.00)	5.00 (2.00, 8.25)	5 (2.00, 8.00)
Number of systems affected median (IQR)	2.00 (1.00, 3.00)	4.00 (2.00, 5.00)	3 (2.00, 5.00)
Asthma (%)	7032 (8.64%)	398 (8.40%)	70 (9.40%)
Obesity (%)	7516 (9.24%)	1008 (21.28%)	220 (29.53%)
Diabetes mellitus (%)	5212 (6.40%)	1197 (25.27%)	214 (28.72%)
Heart failure (%)	646 (0.79%)	343 (7.24%)	33 (4.43%)
COPD (%)	1059 (1.30%)	350 (7.39%)	46 (6.17%)
Arterial hypertension (%)	12,210 (15.00%)	2216 (46.79%)	356 (47.79%)
Depression (%)	7345 (9.02%)	800 (16.89%)	103 (13.83%)
HIV (%)	121 (0.15%)	10 (0.21%)	3 (0.40%)
Ischemic cardiomyopathy (%)	1349 (1.66%)	420 (8.87%)	75 (10.07%)
Stroke (%)	961 (1.18%)	353 (7.45%)	37 (4.97%)
Renal insufficiency (%)	1267 (1.56%)	515 (10.87%)	66 (8.86%)
Cirrhosis (%)	1672 (2.05%)	256 (5.41%)	57 (7.65%)
Osteoporosis (%)	2453 (3.01%)	431 (9.10%)	51 (6.85%)
Osteoarthritis (%)	5212 (6.40%)	854 (18.03%)	121 (16.24%)
Arthritis (%)	1111 (1.37%)	132 (2.79%)	21 (2.82%)
Dementia (%)	859 (1.06%)	296 (6.25%)	9 (1.21%)
Chronic pain (%)	39 (0.05%)	9 (0.19%)	4 (0.54%)
**Outcome**			
Discharge (%)	81,132 (99.69%)	4082 (86.19%)	512 (68.72%)
Deceased (%)	254 (0.31%)	654 (13.81%)	233 (31.28%)

Continuous data are reported as median with interquartile range (Q3–Q1), and categorical data are expressed as percentages (%). COPD is chronic obstructive pulmonary disease and HIV is Acquired Immune Deficiency Syndrome. Deceased (report only when the patient dies).

Three types of patients were identified from the exploratory analysis (Table 1), the COVID-19 most common type of patient that did not require hospitalization was a 38-year-old female (IQR: 22–52) (53.06% of our outpatients are female), with 2 chronic pathologies and 2 affected systems, whose most common pathologies or comorbidities were arterial hypertension (15.00%), followed by obesity (9.24%), depression (9.02%), and asthma (8.64%). In contrast, the prototypical COVID-19 hospitalized non-ICU patient was a 62-year-old male (IQR: 47–79) (51.73% were men), with 5 chronic pathologies, with 4 affected systems, and with more frequent pathologies or comorbidities such as arterial hypertension (46.79%), diabetes mellitus (25.27%), obesity (21.28%), osteoarthritis (18.03%) and depression (16.89%). Finally, the profile of the patient admitted to the ICU was a 62-year-old male (IQR: 52–71) (70.34% of ICU patients were male), with 5 chronic pathologies, 3 affected systems, and whose most frequent pathologies or comorbidities were arterial hypertension (47.79%), followed by obesity 29.53%, diabetes mellitus 28.72%, and osteoarthritis (18.03%). COVID-19 Patients in the ICU had almost twice the possibility of dying than those hospitalized not admitted to the ICU (31.28% vs. 13.81%), far removed from that presented by outpatients (0.31%).

**Figure 2:**
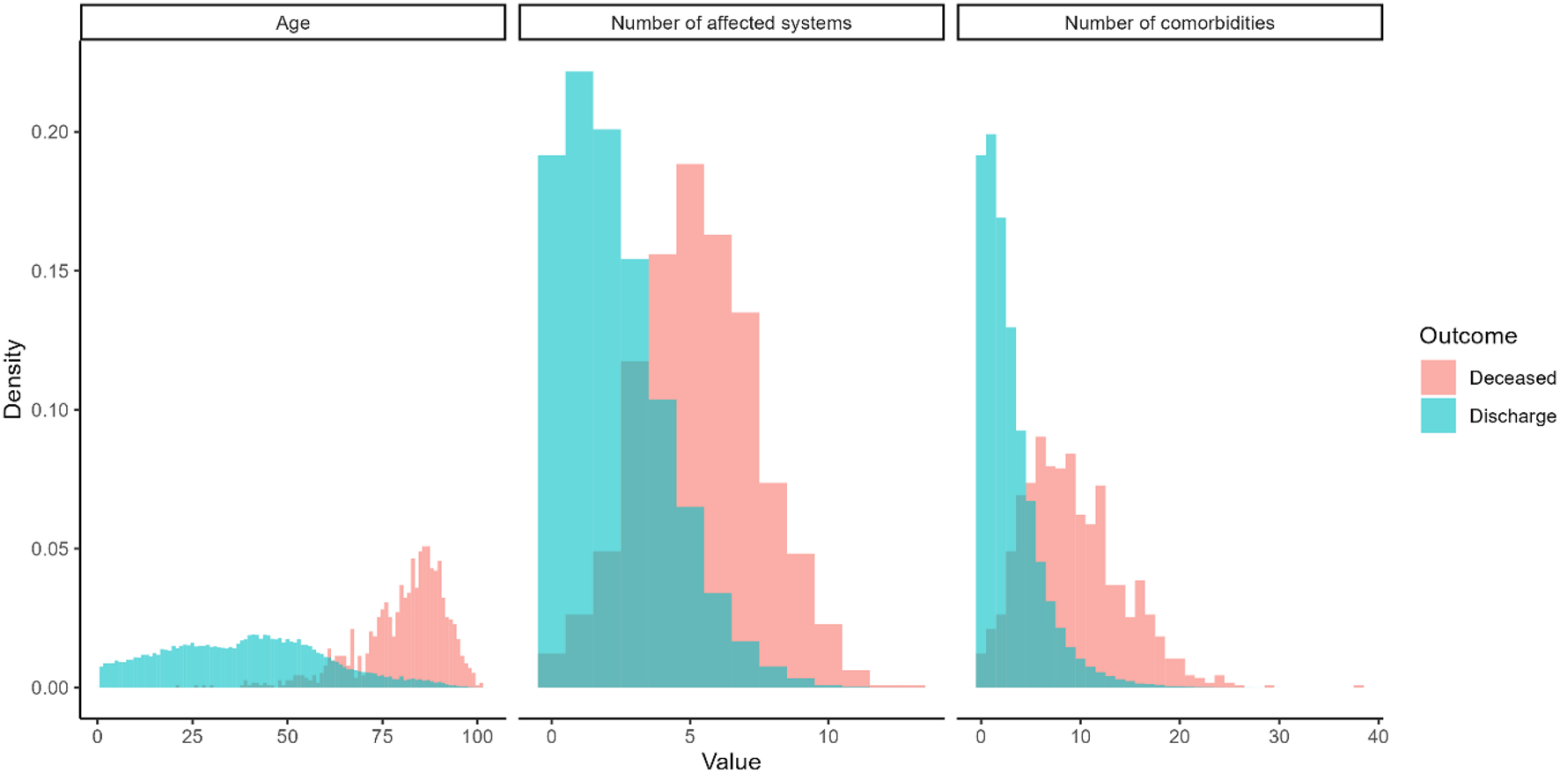
Feature distribution. Distribution of age, the number of affected systems, and the number of comorbidities regarding the final patient outcome. We clearly see different distributions of these variables between discharge and deceased patients.

To further study the differences between discharged COVID-19 patients (survivors) and those who deceased (non-survivors), the data in Table 1 were reorganized in Table 2. The prototype of the surviving patient was a 39-year-old female (IQR: 23–53) (52.72% are female), with 2 chronic pathologies, 2 affected systems, and with more frequent pathologies or comorbidities similar to those previously described for the non-hospitalized patients. In contrast, the profile of the non-surviving patient was clearly different and represented by an 83-year-old man (IQR: 75–88) (56.00% were men), with 8 chronic pathologies, 5 affected systems, and whose prevalent pathology or comorbidity was arterial hypertension (75.64%), far from the subsequent ones such as diabetes mellitus (42.33%), obesity (29.36%), osteoarthritis (27.93%) and depression (23.31%). In addition, three variables were highly relevant to the patient's final status (Fig. 2). Thus, the older the patient *t*(1237) = 116.9, *p* < 2.2 × 10^–16^, the greater the number of chronic pathologies *t*(1151) = 42.15, *p* < 2.2 × 10^–16^ and the greater the number of affected systems *t*(1163) = 47.2, *p* < 2.2 × 10^–16^, the greater the probability of death (Fig. 2). A similar distribution of the above variables was observed when the population was divided into the three initial groups (outpatients, hospitalized non-ICU, and ICU) (Supplementary Fig. S1). The relationship between gender and patient status was significant (X^2^_(1, N = 86867)_ = 34.33, *p* = 4.64 × 10^–9^). Thus, men were more likely to die than women (Table 2, Supplementary Fig. S2), and that the highest risk comorbidities or pathologies were renal insufficiency (OR = 1.90, 95% CI 1.61, 2.24), heart failure (OR = 1.85; 95% CI 1.53, 2.23), stroke (OR = 1.84 95% CI 1.54, 2.20), dementia (OR = 1.78; 95% CI 1.50, 1.81) and ischemic cardiomyopathy (OR = 1.58; 95% CI 1.33, 1.88). However, there was no significant relationship between asthma, osteoarthritis, and osteoporosis with COVID-19–related death.

**Table 2:** Demographic characteristics and comorbidities of discharge and deceased COVID-19 patients.

Characteristics	Discharge (survival)	Deceased (non-survival)	OR (95% CIs)
Number of individuals (N)	85,726	1141	–
Age median (IQR)	39.00 (23.00, 53.00)	83 (75.00, 88.00)	–
**Gender**			
Male (%)	40,535 (47.28%)	639 (56.00%)	2.41 (2.11, 2.75)
Female (%)	45,191 (52.72%)	502 (44.00%)	–
**Comorbidities**			
Number of Chronic diseases median (IQR)	2.00 (1.00, 4.00)	8 (5.00, 12.00)	–
Number of systems affected median (IQR)	2.00 (1.00, 3.00)	5 (4.00, 7.00)	–
Asthma (%)	7403 (8.64%)	97 (8.50%)	1.13 (0.90, 1.42)
Obesity (%)	8409 (9.81%)	335 (29.36%)	1.57 (1.36, 1.81)
Diabetes mellitus (%)	6140 (7.16%)	483 (42.33%)	1.55 (1.36, 1.77)
Heart failure (%)	823 (0.96%)	199 (17.44%)	1.85 (1.53, 2.23)
COPD (%)	1290 (1.50%)	165 (14.46%)	1.49 (1.23, 1.81)
Arterial hypertension (%)	13,919 (16.24%)	863 (75.64%)	1.31 (1.12, 1.53)
Depression (%)	7982 (9.31%)	266 (23.31%)	1.24 (1.06, 1.44)
HIV (%)	134 (0.16%)	0 (0.00%)	–
Ischemic cardiomyopathy (%)	1631 (1.90%)	213 (18.67%)	1.58 (1.33, 1.88)
Stroke (%)	1145 (1.34%)	206 (18.05%)	1.84 (1.54, 2.20)
Renal insufficiency (%)	1591 (1.86%)	257 (22.52%)	1.90 (1.61, 2.24)
Cirrhosis (%)	1923 (2.24%)	62 (5.43%)	1.41 (1.07, 1.86)
Osteoporosis (%)	2758 (3.22%)	177 (15.51%)	1.06 (0.88, 1.29)
Osteoarthritis (%)	5868 (6.85%)	319 (27.96%)	1.03 (0.89, 1.20)
Arthritis (%)	1214 (1.42%)	50 (4.38%)	1.44 (1.05, 1.98)
Dementia (%)	911 (1.06%)	253 (22.17%)	1.78 (1.50, 2.12)
Chronic pain (%)	50 (0.06%)	2 (0.18%)	–

Deceased (report only when the patient dies). Continuous data are reported as median with interquartile range (Q3–Q1), and categorical data are expressed as percentages (%). We also used odds ratio (OR) and 95% CIs. COPD is a chronic obstructive pulmonary disease and HIV is Acquired Immune Deficiency Syndrome.

### ML models

Several machine learning models were developed: (1) to predict the patient’s final condition and (2) to predict which patient will need to be hospitalized. The training dataset (85,476 surviving and 891 non-surviving patients) was used to train the model to predict the final patient’s condition and the test dataset (500 patients; 250 patients from each class) was used to evaluate this model. To realistically evaluate the model, 101 test sets were created with the same proportion as the initial set (1/75), i.e., for each deceased COVID-19 patient, 75 surviving patients were taken.

**Figure 3:**
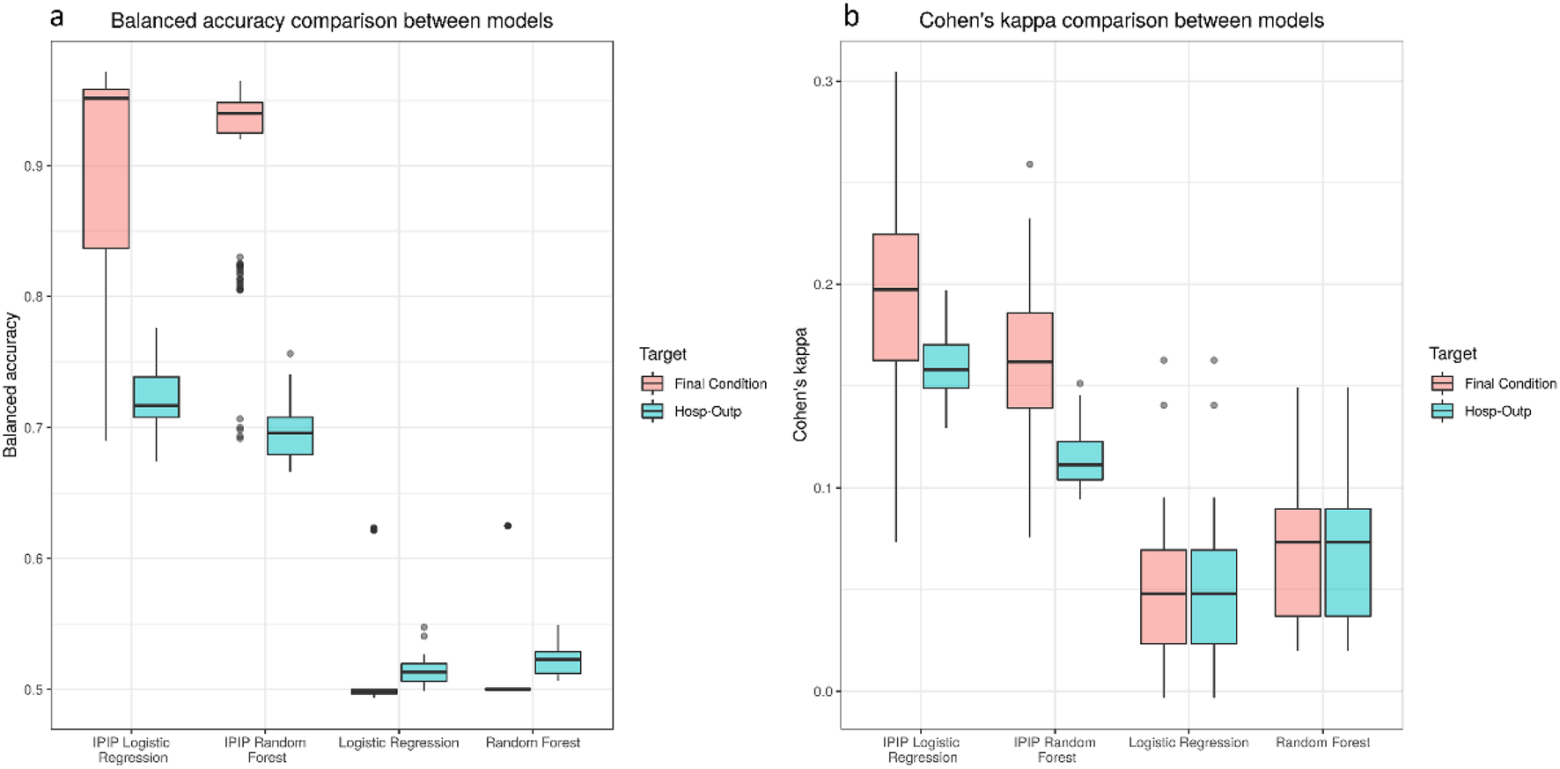
Accuracy and Cohen’s kappa resulting from the different models. (**a**) Accuracy boxplots obtained in the test of the models trained for each problem. (**b**) Cohen’s Kappa coefficients boxplots obtained in the test of the models trained for each problem.

**Figure 4:**
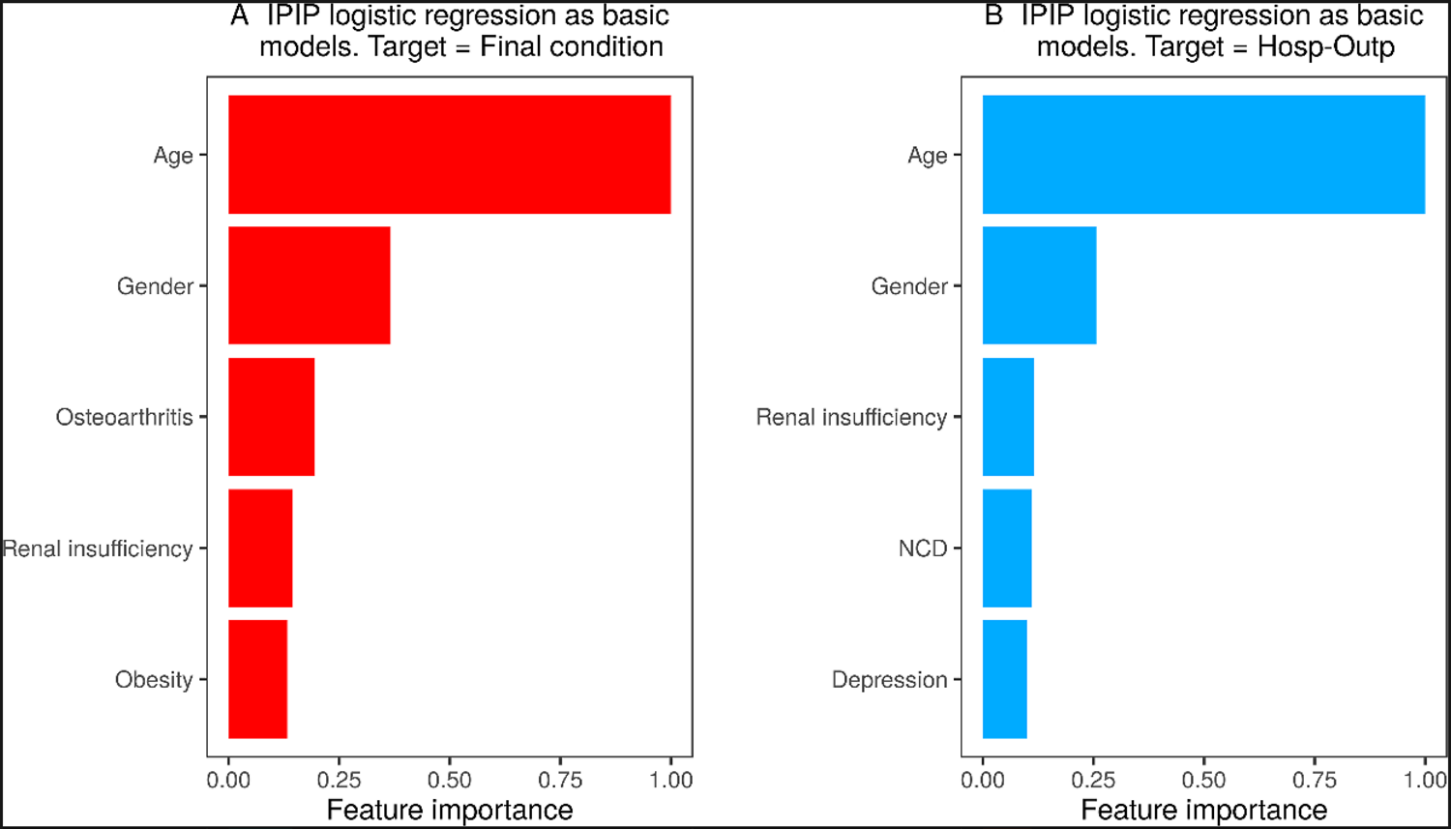
Feature importance. (**a**) The most important factors determining the patient's final condition (Importance Features) obtained by the LR-IPIP model. (**b**) The most important factors determining the need for hospitalization obtained by the LR-IPIP model. NCD is the number of chronic diseases.

Two machine learning algorithms (Random Forest and Logistic Regression) were evaluated with or without IPIP (Identical Partitions for Imbalance Problems), a method to deal with imbalanced data (see “Methods”). The accuracy and Cohen's Kappa obtained on the test data set for the ensemble models (Fig. 3) showed that the IPIP model with logistic regression (LR-IPIP) obtained the best results regarding the final condition of the patient. This LR-IPIP model combined the result of the ensemble of 21 logistic regression models. The patient's final condition can be predicted with the LR-IPIP model with a balanced accuracy between 0.90 and 0.93 (Table 3) for the imbalanced datasets versus 0.91 for the balanced datasets, balanced datasets are the ones usually used in the literature and which give a higher Cohen's Kappa coefficient (0.83 vs. 0.20). In addition, the ROC-AUC of this model for the imbalanced datasets was 0.94 (Supplementary Fig. S3a). The most important factors determining the patient's final condition (Importance Features) obtained by this LR-IPIP model were firstly age (FI: 1.0), followed by sex (FI: 0.366), osteoarthritis (FI: 0.194), renal insufficiency (FI: 0.144), obesity (FI: 0.132) and the number of affected systems (FI: 0.117) (Fig. 4a, Supplementary Fig. S4).

**Table 3:** Metrics obtained in the test dataset using the best training model.

Metrics	Deceased/discharge		Hospitalized/outpatient	
	Imbalanced tests	Balanced test	Imbalanced tests	Balanced test
Balanced accuracy	0.92 (0.90, 0.93)	0.91	0.72 (0.71, 0.73)	0.72
Cohen’s Kappa	0.20 (0.18, 0.21)	0.83	0.16 (0.15, 0.17)	0.44
Sensitivity	0.93 (0.88, 0.96)	0.92	0.72 (0.70, 0.74)	0.71
Specificity	0.91 (0.90, 0.91)	0.91	0.73 (0.72, 0.73)	0.73
Positive predictive value (PPV)	0.12 (0.11, 0.13)	0.91	0.15 (0.14, 0.15)	0.72
Negative predictive value (NPV)	1 (0.99, 1)	0.92	0.98 (0.97, 0.98)	0.72

Balanced tests are sets with the same number of patients of each class. Imbalanced tests are sets with the same proportion of classes as the original dataset, where the number of examples for each class label is not balanced. Those sets are made up by a chosen number of samples of patients for each class. For imbalanced tests we report the mean (95% CIs) of each metric, and for balanced tests we report the value of each metric.

On the other hand, the model to predict which patient will need to be hospitalized was developed using a training dataset (4385 inpatients and 80,290 outpatients), and a test dataset (2192 patients; 1096 patients from each class) was used to evaluate this model. In turn, this data was distributed in 25 test sets with the same ratio of inpatients/outpatients as in the initial set (1/15). Again, the model with the best results was the LR-IPIP made up of 13 logistic regression models (Fig. 3). The need for hospitalization could be predicted with the LR-IPIP model with a balanced accuracy between 0.71 and 0.73 (Table 3) for the imbalanced datasets versus 0.72 for the balanced datasets. Similar to the other model, the Cohen's Kappa coefficient is higher for the balanced dataset (0.44 vs. 0.16). In addition, the ROC-AUC of this model for the imbalanced datasets were 0.746 (Supplementary Fig. S3b). Finally, the significance of the characteristics obtained in that model showed that age (FI: 1.0) was also the most relevant characteristic, followed by sex (FI: 0.26), renal insufficiency (FI: 0.12), number of chronic diseases (FI: 0.11) and depression (FI: 0.1) (Fig. 4b, Supplementary Fig. S4).

## Discussion

In this study, we have analyzed the different COVID-19 patient types in Southeastern Spain (n = 86,867). In contrast to most COVID-19 studies that developed predictive models in the literature that handle less than 5000 patients^17–21^. In addition, we have presented a technique specially designed to treat imbalance problems (IPIP), with which we have developed machine learning models to predict the final state of the patient and the need for hospitalization of those. We trained and evaluated the models with and without IPIP, which efficiently manages the imbalance in the data according to our results (Fig. 3).

Regarding characterizing the different kinds of prototypical COVID-19 patients, in this region, the COVID-19 most common type of patient that did not require hospitalization is a 38-year-old woman, with 2 chronic pathologies whereas the hospitalized COVID-19 patient prototype is a 62-year-old man, with 5 chronic pathologies. We identified age, gender, and the number of comorbidities as important to distinguish between outpatient and hospitalized. Several studies have also found that hospitalized COVID-19 patients are more commonly older, male, and associated with more comorbidities such as obesity, diabetes mellitus, and hypertension^22,23^. In addition, we could find statistically significant differences for age (*p* < 8.0 × 10^–3^), the number of comorbidities (*p* < 2.5 × 10^–3^), and gender (*p* < 2.2 × 10^–16^) between ICU and hospitalized non-ICU patients, although those differences are smaller than between outpatient and hospitalized. ICU patients were around a year younger than hospitalized non-ICU patients and had fewer comorbidities (Supplementary Fig. S1). Therefore, we hypothesized that clinicians included patients more likely to survive in the ICU because of the limited number of available ICU slots or the risk of the male gender. We also detect even more differences for those features between survivors (discharge patients) and non-survivors (deceased patients) (Fig. 2). In our region, the discharge patient prototype is a 39-year-old woman, with 2 chronic pathologies while the deceased patient prototype is an 83-year-old man, with 8 chronic pathologies. According to several studies, our results show that older patients are more likely to die^24–26^, and also male patients are more likely to die (OR = 2.41, 95% CI 2.11, 2.75) (Table 2, Supplementary Fig. S2)^27,28^. When it comes down to comorbidities, we found that asthma, osteoporosis, and osteoarthritis are not associated with COVID-19–related death. A large number of studies report that patients with asthma are not at risk of severe COVID-19^29,30^. For osteoarthritis association with COVID-19–related death we found a study that reported similar OR = 0.84 (95% CI 0.65–1.08)^31^. For osteoporosis, it is known that women are more at risk of developing osteoporosis than men^32^. It seems that some particular kinds of osteoporosis complications are associated with more risk of COVID-19 exitus, however, this study did not adjust the risk by age and gender^33^. The rest of the comorbidities evaluated in our study were associated with an increase in mortality risk. These comorbidities or pathologies are diabetes mellitus, dementia, obesity, heart failure, COPD, arterial hypertension, ischemic cardiomyopathy, stroke, renal insufficiency, cirrhosis, and arthritis. Several studies obtain the same results for those comorbidities^31,34,35^. Regarding depression, in line with our results, a meta-analysis identified that depression is associated with more COVID-19-related death^36^. All the results mentioned above are important to ensure that the characteristics and comorbidities of our population were not unique. In addition, we believe that due to the similarity with other COVID-19 studies our data could be useful to develop predictive models.

Since the beginning of the pandemic, there have been many studies that have reported some important clinical characteristics (predictors) for mortality in patients with COVID-19 through the development of ML-based models. Selected characteristics used as inputs for the development of these models included baseline data, clinical symptoms, associated comorbidity, and clinical indicators. However, these studies have two fundamental problems: the low number of patients due to the number of parameters studied greatly restricts the cohort and the strongly imbalanced data. To bridge these drawbacks, in this work we tested different ML models considering basic data easily accessible in an emergency care setting and based on clinical data from EHR to help during early patient triage. We definitely obtained promising results when predicting the patient's final condition using the LR-IPIP model (0.92 balanced accuracy, ROC-AUC = 0.94). In terms of variable importance, ML detects Age (FI: 1.0), gender (FI 0.366), osteoarthritis (FI: 0.194), renal insufficiency (FI: 0.144), obesity (FI: 0.123), and the number of systems affected (FI: 0.117) as the most important variables to predict *exitus*. The model also detected comorbidities such as dementia, diabetes mellitus, and COPD. These features are associated with more risk of COVID-19–related death according to our model. In a similar direction, these comorbidities are associated with severe clinical manifestations observed in older adult patients^37,38^. Comorbidities such as cardiovascular disease, hypertension, and diabetes although are highly prevalent in older adults have been associated with worse outcomes in COVID-19^31,34,35^. Studies that rely on comorbidities to predict death based on ML usually rank age as one of the most influential variables^39,40^, in fact, a meta-analysis with 611,583 patients demonstrates an age-related increase in mortality. Thus, the highest mortality occurs in patients > 80 years, in whom it was 6 times higher than in younger patients^41^. Similarly, gender is an important feature for several ML-based studies^39,42^, our model identified that male patients are more likely to die, perhaps due to the distribution of our data (OR = 2.41, 95% CI: 2.11, 2.75), which is in agreement with previous work^27,28^. Similar to our model, another ML-based study identified obesity as an important feature^43^. However, to the best of our knowledge, this is the first time that a model reports osteoarthritis as an important feature. The beta values in the ensemble model showed that osteoarthritis is associated with less risk of COVID-19–related death (Supplementary Table S2). This might be in agreement with a study using UK biobank data (OR = 0.84, 95% CI 0.65–1.08), although it is not statistically significant^31^. In addition, the osteoarthritis distribution in our population is not statistically associated with the patient's final condition. Note that, although we have no conclusive evidence on this, patients with osteoarthritis may be subjected to medication. Interestingly, we might think that medication could play a role in patients with osteoarthritis and COVID-19, however, Wong et al. reported that non-steroidal anti-inflammatory drugs (NSAIDs) medication is not associated with a higher risk of COVID-19 death for osteoarthritis patients^44^. Dementia, together with the number of affected systems and the number of comorbidities, also appear among the most relevant characteristics, which is in agreement with the aforementioned factors in other studies, and in the case of dementia, with the results obtained from a cohort of 12,863 individuals from the UK Biobank who lived in the community and were over 65 years of age (1814 individuals ≥ 80 years of age) were tested for COVID-19, where it was seen that all causes of dementia increased the risk of death related to COVID-19^45^. Regarding accuracy, our LR-IPIP model obtained a balanced accuracy between 89 and 93% (ROC-AUC = 0.94) in predicting the patient's final condition. Accuracy was similar to or higher than others if we compare our results with several studies. For instance, Gao et al. reported an accuracy between 80.6 and 96.8%^18^ which is a large confidence interval besides they used more complex clinical data points on admission. Chatterjee et al. reported a balanced accuracy of 72%^20^, perhaps due to the low number of COVID-19 patients. Finally, another ML-based study was able to predict the risk of death already at diagnosis with a ROC-AUC of 0.902^21^.

The ability of the LR-IPIP model to decide the hospitalization of new patients was not as efficient (balanced precision = 0.72; ROC-AUC = 0.75). Regarding the importance of the variables, ML again found that age, gender, and the number of comorbidities were important. Among these, obesity reappears, and renal insufficiency and depression appear in a prominent place. Thus, it has been shown that acute renal failure is frequent among patients hospitalized for COVID-19 and that only 30% survived with the recovery of renal function at discharge^46^.

This research has specific shortcomings. Firstly, due to the highly specific character of this cohort and its unavoidable novelty, we were unable to easily obtain an alternative cohort that may be used for replication and validation of our findings. Fortunately, this was partially overcome by the fact that individuals came from a variety of hospitals in our region with shared data management of electronic health records. As this was a retrospective study, the lack of some data was compensated for by including in the study only those demographic data and comorbidities that had been correctly recorded. Secondly, another difficulty stems from the strong data imbalance inherent in the research question we make. We tried to compensate this with the development of the IPIP method. Thirdly, it must be noted that the data used to build the models was obtained in the absence of vaccination patterns and new variants of the SARS-COV-2 virus. However, the methodology to build the models can be easily adapted to these new scenarios. Finally, a better understanding of the contribution of different symptoms or comorbidities to disease diagnosis could serve to introduce new features in future models, especially to improve the prediction of patients who do or do not require hospitalization.

In conclusion, this paper shows the analysis and development of predictive ML-based models with one of the largest COVID-19 datasets (n = 86,867) obtained from the health service of the Region of Murcia (Spain). In addition, the problem of class imbalance has been addressed by developing a new algorithm, called IPIP, which automatically deals with this problem. The model obtained allows predicting with high accuracy the final state of the patient, and with reasonable precision which patient will need to be hospitalized, simply by using the demographic data and comorbidities accessible at COVID-19 diagnosis by the clinicians. In fact, this LR-IPIP predictive model can be used, among other considerations, to prioritize triage of COVID-19 patients when health system resources are limited, as is often the case during different waves of COVID-19. To facilitate this prioritization of resources, both the corresponding web application and the predictive models are easily accessible in open repositories (GitHub), which will facilitate their adaptation to new datasets of future epidemic waves of this disease or other respiratory viruses in general.

## Methods

### Study design and participants

Patients who were diagnosed with COVID-19 consecutively enrolled between January 4, 2020, and February 4, 2021, comprised our cohort. A confirmed case with COVID-19 is defined as a positive result of antigen test or real-time reverse-transcriptase polymerase-chain-reaction (RT-PCR) assay for nasal and pharyngeal swab specimens. Patients who were excluded from the "patient stratification" table are patients either with an unavailable characteristic (6192 patients), or with still active COVID-19 disease at the closing date of patient recruitment (9513 patients), or patients with erroneous values that could not be possible (1 patient). The data in our analysis and models included 86,867 confirmed COVID-19 patients (Fig. 1). Features included in our study derived from the "patient stratification" database which includes information for each patient about age, sex, diabetes mellitus, dementia, obesity, heart failure, chronic obstructive pulmonary disease (COPD), asthma, arterial hypertension, depression, ischemic cardiomyopathy, stroke, renal insufficiency, cirrhosis, osteoporosis, osteoarthritis, arthritis, Acquired Immune Deficiency Syndrome (VIH), and chronic pain.

### Access to personal data

The information of interest for the study has been obtained from the files of medical records of the Murcia Health Service in Spain, without the consent of the holders of the data. The need for informed consent was waived by the Bioethics Committee of Universidad de Murcia. This was done in accordance with the following criteria:

- Article 157 of the General Data Protection Regulation (RGPD) recognizes the benefit that access to records can provide to research on diseases, so that the results of these studies could be more solid, based on a larger population.
- Article 89.1 of the RGPD confirms this principle as long as the appropriate measures are adopted, in particular respect for the principle of minimization of personal data. These measures may also include the use of pseudonymized or anonymized data.
- In turn, article 14.5 b) of the RGPD allows the data controller not to inform the interested parties when "said information is impossible or involves a disproportionate effort in particular for the treatment for purposes of public interest, scientific research purposes or historical or statistical purposes subject to the conditions and guarantees indicated in article 89, paragraph 1, or to the extent that the obligation mentioned in paragraph 1 of this article may make it impossible or seriously impede the achievement of the objectives of such treatment”.
- The seventeenth additional provision of Organic Law 3/2018, of December 5, on the Protection of Personal Data and guarantee of digital rights allows the use of health data for scientific research purposes as long as these data have been submitted to prior treatment of pseudonymization or anonymization.

In accordance with the exposed precepts, consent is not necessary for the following reasons:

1. Access to clinical records for scientific research purposes is lawful, as long as certain guarantees are adopted, including respect for the principle of data minimization and that the information obtained is adequately pseudonymized or anonymized.
2. Obtaining consent would not be a legal requirement, since it would not only entail disproportionate efforts but could also hinder the development of the study due to the large amount of information that has been analyzed.
3. The Murcia Health Service, in accordance with the provisions of the aforementioned regulations, has submitted this clinical information to an anonymization process, excluding any identifying information of the patients, making their identification impossible.

### Data description and preprocessing

Epidemiological and clinical data for COVID-19 were collected from electronic medical records of the Regional Health System (SMS) from the Region of Murcia (Spain) and their use for this work and within all the experiments therein was approved by the Ethics Committee of the University of Murcia. Therefore, all experiments were performed in accordance with relevant guidelines and regulations. The database stratifying patients diagnosed with COVID-19 (a total of 102,573 patients) includes age, sex, hospital, and primary care unit assigned to the patient, admission information and final status (i.e., the patient is cured or has deceased), information on comorbidities, number of chronic pathologies and number of systems affected, as well as risk stratum. Other data limiting the number of patients, such as hospital dispensing medication (9165 patients), length of stay in each department (8356 patients), information on vital signs (7524 patients) or information on various symptoms presented by 89,769 patients (headache, fever, dizziness, vomiting, among others) were not used in order to simplify the data necessary for triage of patients in situations of hospital system collapse during a pandemic outbreak.

### Prediction models

We use machine learning to develop models about questions of interest: what the final condition of the subject will be and whether the individual will be hospitalized. We want to be able to answer these questions when the patient has just been diagnosed, therefore the only information that we can use for that is obtained in a medical review or the previous information available in the EHR of the patient. In this study, what we have at our disposition is a table that contains information on the age, sex, comorbidities, hospitalization status, and final outcome of the patients. The available datasets for both questions are highly imbalanced, as there are far fewer deceased individuals (1141) than discharged patients (85,726), and there are more outpatients (81,386) than hospitalized (non-ICU 4736 and ICU 745). To address these issues, we propose a new imbalance-aware and ensemble-based machine learning method. It is called IPIP (Identical Partitions for Imbalance Problems). First, we hold out 20% of the data from the minority class and the same number of samples from the majority class to create a test set. The rest goes into a train set. We then divide the training data set into *p* balanced data sets. This requires setting up a hyperparameter of the algorithm, called variability of the proportion. By default, IPIP creates p perfectly balanced (50–50%) datasets for binary classification problems. This hyperparameter allows to specify a random interval of variability of that proportion. For example, a value of 0.05 randomly increments, from 50% up to a 5% more, the proportion of the majority class for each of the p resamples. In this particular problem, the proportion variability hyperparameter was set up to 0.05 for predicting the final condition. And for the hospitalization problem we used perfectly balanced datasets. These final values were obtained by empirically testing the range of values (Supplementary Fig. S5) how IPIP models behave at a range of proportion variability values. Once the p resamples are created, for each, we create a basic ML model. All models go into an ensemble whose response aggregation is a simple majority. IPIP selects *p* depending on *n* (number of samples). Higher values of *n* lead to lower values of *p*. For this particular dataset, 75% of the minority class samples of the train data leads to p = 7 subsets. Within each *p* iteration, it randomly splits into train and test datasets and generates a model with training data and testing with test data. If the new model improves the overall quality of the ensemble, it is added. If not, it randomly samples and tries again up to a max number of attempts. We use the test set to evaluate the candidate ensemble for improvement. When doing inference, the classifier predicts an observation as a member of the majority class when at least 75% of the models classified as negative (majority class) will be classified as negative. The final ensemble´s inference in production mode is generated by evaluating each one of the models that compose the final ensemble, if 50% of them classify a sample as negative (majority class), the final model classifies it as negative. Predictive models found at the literature are based on a variety of models: ensembles of decision trees^17^, support vector machine classifiers, neural networks^18^ or just simply logistic regression lasso models^19^. The IPIP model is an ensemble composed of a set of basic logistic regression models created from different subsamples of the original data. The overall classifier predicts an observation as a member of the majority class when at least 75% of the models classified as negative (majority class) will be classified as negative. Therefore, the IPIP models are extremally efficient both in terms of computing and storage requirements.

We created two IPIP models to address each modeling question. One with a baseline algorithm, logistic regression, and the other with random forests^47^ as basic models based on the Caret R package^48^. All models were evaluated using five-fold cross-validation. Different values of the number of decision trees for the random forest models were tested, and we decided that each random forest model used 200 decision trees, where the impurity is the variable importance mode, which is the Gini index for classification. A tune grid was created to choose the better minimal node size of the trees (1, 11, or 21) and the number of variables to possibly split into each node (1, 4, 7, 10, 13, 16, or 19). To decide whether a basic model improves the ensemble or not, we relied on Cohen’s Kappa metric^49^. That is, if adding a basic model to the set of basic models trained for a specific perfectly balanced subset improved the Kappa in the evaluation on the available test set of the new set of basic models concerning the previous Kappa values on the same evaluation set, we added that basic model to that set of basic models, otherwise it was discarded. We also obtained the following metrics: balanced accuracy, negative predictive value (NPV), positive predictive value (PPV), sensitivity, and specificity. In addition, we computed the Receiver Operating Characteristics Area Under the Curve (ROC-AUC) for the final ensemble model.

### Statistical analysis

Continuous data are reported as median with interquartile range (IQR), and categorical data are expressed as percentages (%). We also used odds ratio (OR) and 95% CIs. We adjusted OR by gender and age. Differences between groups were tested using the Mann–Whitney U for numerical variables, and χ2 test or Fisher’s exact test was used to test significance for categorical data. Statistical analysis was performed by using R (version 3.6.3) with P-values significance threshold of 0.05.

## Supplementary Information

Supplementary Information.

## Data Availability

All the data used in this study were retrieved from the Servicio Murciano de Salud (SMS). All data produced in the present study are available upon reasonable request to the authors and the approval by Servicio Murciano de Salud (SMS). We developed a shiny app to share our model and to predict patient hospitalization https://alejandrocisterna.shinyapps.io/PROVIA.

## Code availability

The code required to reproduce the models and results are available at: https://github.com/antoniogt/ipip.
