# Supplementary material for: A predictive model for hospitalization and survival to COVID-19 in a retrospective population-based study
Source: Sci Rep. 2022 Oct 28;12:18126. doi: 10.1038/s41598-022-22547-9 (PMC9614188; doi:10.1038/s41598-022-22547-9)
Supplement: Supplementary file 1 — Supplementary Information. [file 41598_2022_22547_MOESM1_ESM.pdf]

Supplementary figures and tables

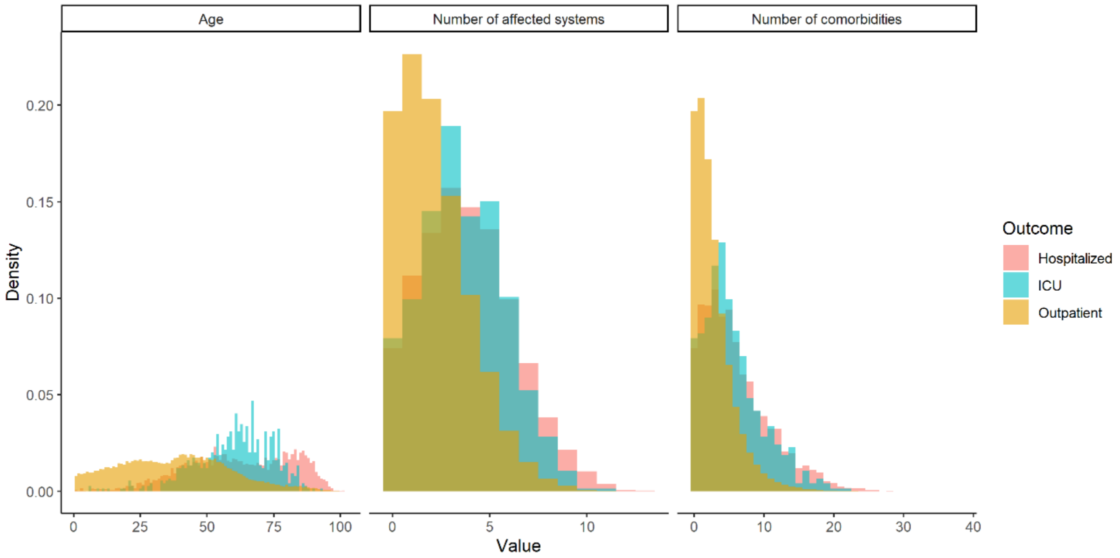

Supplementary Figure S1. Distribution of age the number of affected systems and the number of comorbidities regarding the type of patient. We clearly see different distributions of these variables between outpatient (patients that did not require hospitalization) and hospitalized (ICU and non-ICU) patients.

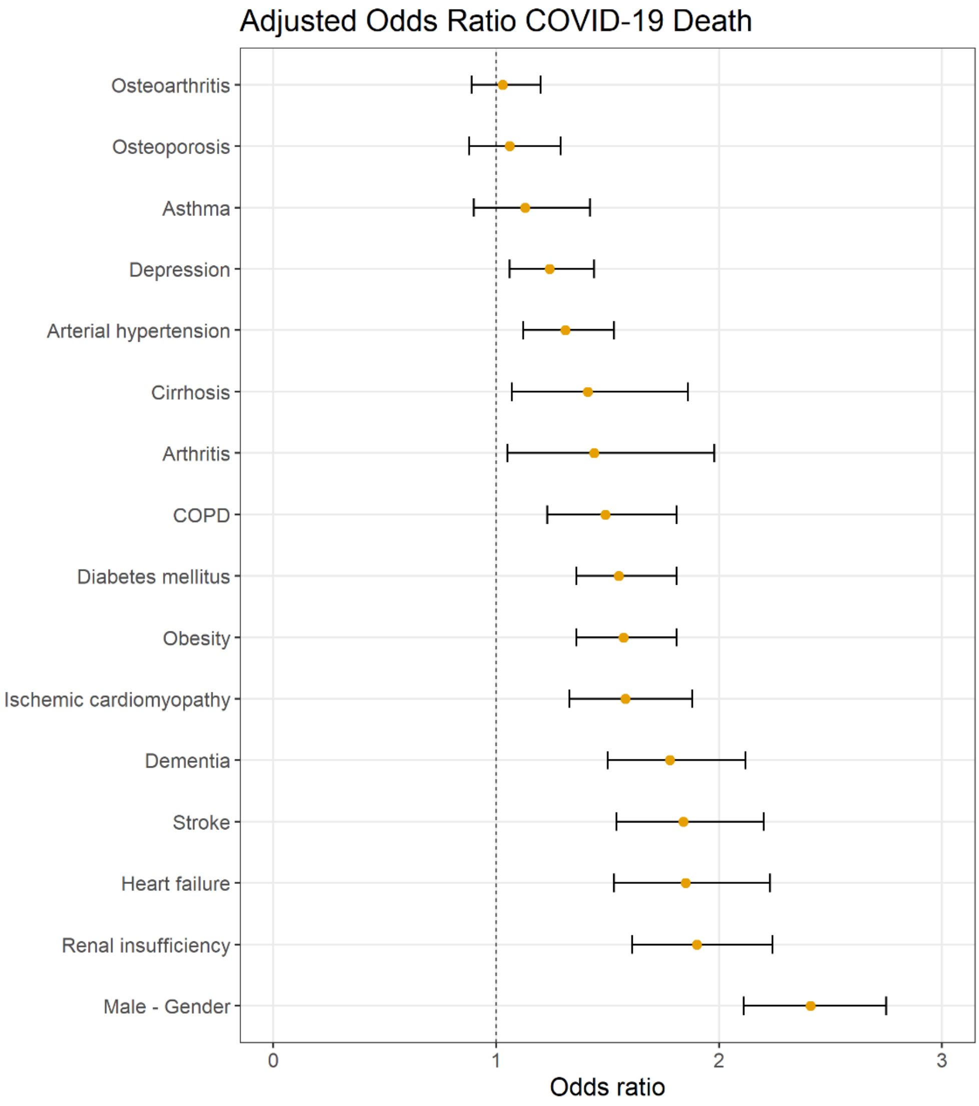

Supplementary Figure S2. Adjusted Odds Ratio COVID-19 Death

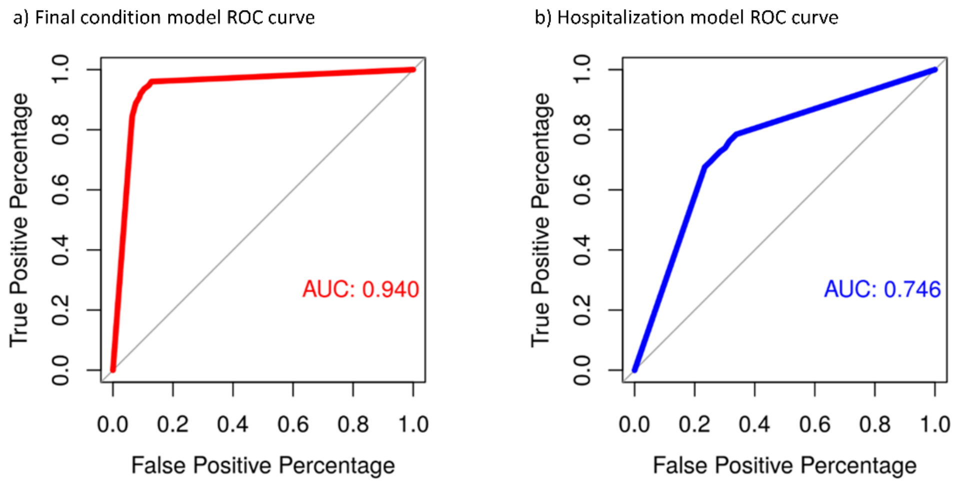

Supplementary Figure S3. ROC curves for final condition (a) and hospitalization (b) predictive models.

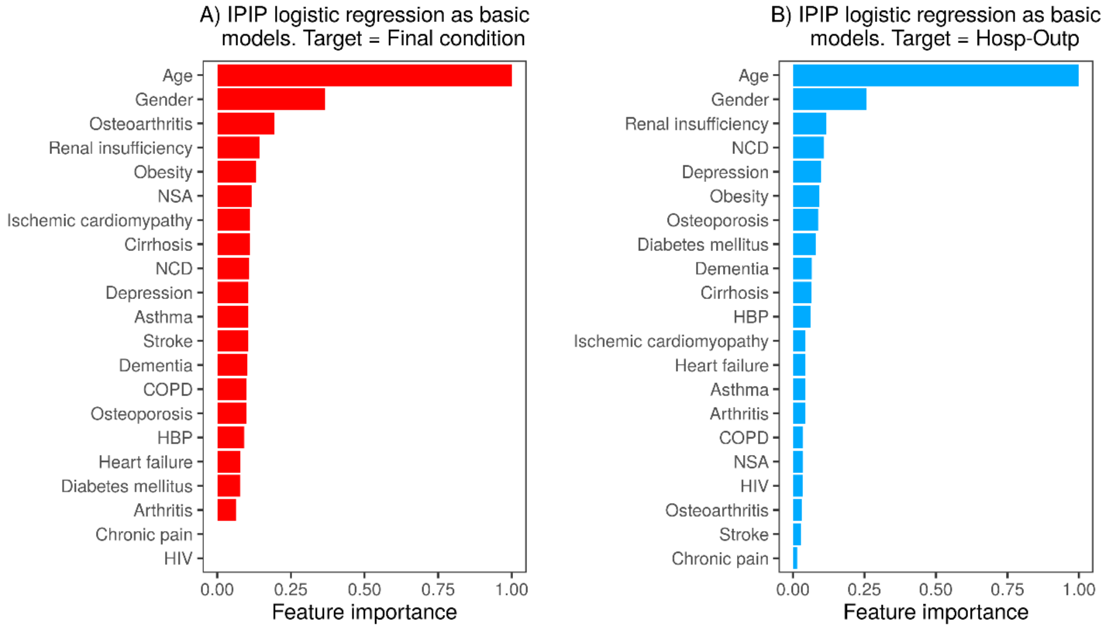

Supplementary Figure S4. Features importance for final condition (a) and hospitalization (b) predictive models.

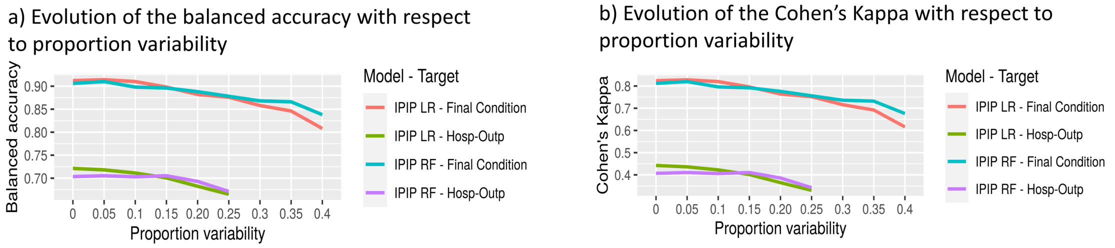

Supplementary Figure S5. Effect of proportion variability values in accuracy and Cohen's Kappa. a) Evolution of the balanced accuracy with respect to proportion variability. X-axis indicates different proportion variability values and y-axis indicates the accuracy obtained. Red and blue curves indicate the results obtained by IPIP model with logistic regression and random forest, respectively, for the final condition problem. Green and purple curves indicate the results obtained by IPIP model with logistic regression and random forest for the hospitalization problem respectively. b) Evolution of the Cohen's Kappa with respect to proportion variability. Same plot but y-axis indicates the Cohen's Kappa obtained. Note that curves in both plots, which correspond to the hospitalization problem end at 0.25 variability because due to how IPIP generates the resamples, it is neither possible nor required to generate p resamples with the features IPIP requires and imbalance higher than 25%.

Supplementary Table S1. Symptoms and its frequency in COVID-19 patients.

**Symptoms** (Number of patients and percentages %)	
Number of individuals	89768
Cough<br>(%)	44875<br>(49.99%)
Headache<br>(%)	34419<br>(38.34%)
Myalgia<br>(%)	32347<br>(36.03%)
Hyposmia<br>(%)	26934<br>(30.00%)
Rhinorrhea<br>(%)	26634<br>(29.67%)
Hypogeusia<br>(%)	24412<br>(27.19%)
Nasal congestión<br>(%)	23677<br>(26.38%)
Sore throat<br>(%)	22186<br>(24.71%)
Expectoration<br>(%)	11372<br>(12.67%)
Shivering<br>(%)	10674<br>(11.89%)
Fever<br>(%)	9810<br>(10.93%)
Chest pain<br>(%)	7121<br>(7.93%)
Abdominal pain<br>(%)	6680<br>(7.44%)
Dizziness<br>(%)	6115<br>(6.81%)
Vomits<br>(%)	3540<br>(3.94%)
Arterial hypertension<br>(%)	2442<br>(2.72%)
Skin problems<br>(%)	1556<br>(1.73%)
Eye problems<br>(%)	1358<br>(1.51%)

Categorical data are expressed as percentages (%).

Supplementary Table S2. Beta values for osteoarthritis for each of 21 logistic regression models (Final condition ensemble).

**Atributes**	**IPIP Logistic Regression Models. Target: Final Condition**																				
	1	2	3	4	5	6	7	8	9	10	11	12	13	14	15	16	17	18	19	20	21
Osteoarthritis – Yes	-0.222	-0.661	-0.612	-1.025	-0.146	-1.088	-0.570	-0.819	-0.734	-0.598	-0.822	-1.265	-0.997	-1.327	-0.224	-0.957	-1.492	-0.905	-0.503	-0.389	-0.611
